# A New Phospholipase D from *Moritella* sp. JT01: Biochemical Characterization, Crystallization and Application in the Synthesis of Phosphatidic Acid

**DOI:** 10.3390/ijms231911633

**Published:** 2022-10-01

**Authors:** Fanghua Wang, Xuejing Mao, Fuli Deng, Ruiguo Cui, Lilang Li, Siyu Liu, Bo Yang, Dongming Lan, Yonghua Wang

**Affiliations:** 1 School of Food Science and Engineering, South China University of Technology, Guangzhou 510640, China; 2 School of Bioscience and Bioengineering, South China University of Technology, Guangzhou 510006, China

**Keywords:** phospholipase D, phosphatidic acid, crystal structure, *Moritella* sp. JT01, monolayer technology, maximum insertion pressure, biphasic reaction system

## Abstract

A new phospholipase D from marine *Moritella* sp. JT01 (MsPLD) was recombinantly expressed and biochemically characterized. The optimal reaction temperature and pH of MsPLD were determined to be 35 °C and 8.0. MsPLD was stable at a temperature lower than 35 °C, and the t_1/2_ at 4 °C was 41 days. The crystal structure of apo-MsPLD was resolved and the functions of a unique extra loop segment on the enzyme activity were characterized. The results indicated that a direct deletion or fastening of the extra loop segment by introducing disulfide bonds both resulted in a complete loss of its activity. The results of the maximum insertion pressure indicated that the deletion of the extra loop segment significantly decreased MsPLD’s interfacial binding properties to phospholipid monolayers. Finally, MsPLD was applied to the synthesis of phosphatidic acid by using a biphasic reaction system. Under optimal reaction conditions, the conversion rate of phosphatidic acid reached 86%. The present research provides a foundation for revealing the structural–functional relationship of this enzyme.

## 1. Introduction

Phosphatidic acid (PA), featuring two fatty acyl chains at the *sn*-1 and *sn*-2 positions and a phosphate group at the *sn*-3 position of the glycerol backbone, is the simplest and a minor class of glycerophospholipids [1]. Physiologically, PA is a key intermediate in lipid metabolism as well as a signaling messenger involved in a variety of metabolic, cellular, and physiological processes, ranging from microbes to mammals and higher plants [2,3]. Considerable progress has been made recently on the production, cellular function, and mode of action of PA in the cell [3,4,5,6,7]. Beyond its biological importance, PA has drawn considerable interest and demonstrated an excellent application potential in food, pharmaceuticals, and other industries. Based on its hydrophilic head and hydrophobic tail, PA can be used as an emulsifier and a drug carrier and is widely used in the food and pharmaceutical industries. For instance, PA can be safely used to mask the bitterness of drugs and foods without affecting the taste responses to other taste stimuli such as salts, acids, and sugars [8]. In the pharmaceutical industry, PA is used as a drug to treat mental stress, depression, and cancer. It is also an active ingredient in medications including antianxiety medications and joint lubricants [9,10,11,12]. The ability of PA to increase muscle strength during training regimens has also been studied as a dietary supplement, which is significant for various population groups (athletes, the elderly, etc.) [13]. More interestingly, PA is an effective component of smoking cessation agents, and its concentration in these agents is 7% [11].

Due to the importance of PA in various industries, the production of PA has become one of the main concerns for researchers. Natural sources of PA include barley germ, corn germ, soy, and egg yolk. However, the content of PA is usually quite low (less than 5%) [2]. Although PA can be prepared via a variety of methods, including thin layer chromatography and solvent extraction, these methods involve complex steps and a large number of solvents, which are harmful to the environment. The most promising method is enzymatic conversion for the synthesis of PA, which is easy to operate and environmentally friendly. Phospholipase D (PLD, EC 3.1.4.4) is a kind of enzyme that specially hydrolyzes naturally abundant phosphatidylcholine [14] to generate PA along with choline, thus becoming the main tool for the enzymatic synthesis of PA [15]. PLDs are ubiquitously distributed in various organisms including mammals, plants, and microorganisms [16]. PLD belongs to the PLD superfamily. Members in the PLD superfamily contain one or two highly conserved HxKxxxxDxxxxxxGG/S (HKD) motifs, which are assumed to act as active centers [17]. Until now, there have been few reports on the crystal structure of PLDs. The resolved structure of PLDs includes bacterial PLDs originating from *Streptomyces* sp. PMF (PMF PLD) [18] and *Streptomyces antibioticus* (SaPLD) [19], and eukaryotic PLDs originating from *Homo sapiens* (PLD1, PLD2) [20,21] and *Arabidopsis thaliana* PLDα1 [22]. In our previous studies, the crystal structure of a PLD from the plant-associated bacteria *Serratia plymuthica* Strain AS9 was resolved, revealing a unique arrangement of catalytic pocket [23]. Although some achievements have been made, the relative scarcity of structural information has greatly hindered the in-depth understanding of the structural–functional relationship of PLDs. Moreover, most research on PLD mainly focuses on the application of its transphosphatidylation activities to the synthesis of rare phospholipids (such as phosphatidylserine, PS) or unnatural phospholipids [16], and few studies report on the enzymatic synthesis of PA by using this enzyme.

In the present study, we first report a new phospholipase D from *Moritella* sp. JT01 (MsPLD). The biochemical and structural properties of MsPLD are characterized in detail. Furthermore, the functions of its unique extra loop segment on the enzyme properties are characterized by using an enzymatic assay and monolayer technology. Finally, the application of MsPLD in the synthesis of PA is investigated. The present research not only lays a foundation to understand the structural–functional relationship of this enzyme but paves a way for the further application of MsPLD in the food and other industries.

## 2. Results

### 2.1. Bioinformatic Analysis of MsPLD

MsPLD had a total of 578 amino acids, and using the online tool SignaIP v. 5.0 web server (http://www.cbs.dtu.dk/services/SignalP/) (accessed on 5 March 2021), the first 28 amino acids of MsPLD was predicted to be the signal peptide (MKIQNKHAMIILKSAASAFVLVSASASA). Two conserved HKD motifs (HXK(X)_4_D(X)_6_GG/S) that are thought to work as active centers of PLDs were also observed in MsPLD while conducting multiple alignment analyses (Figure S1A). The protein sequence identity between SpPLD, PMF PLD, and SaPLD was remarkably low (less than 30%), except the two conserved HKD motifs, and there were no substantial sequence similarities between them (Figure S1B). Despite this, the presence of highly conserved motifs in the protein sequence and subsequent specific activity assays clearly suggested that MsPLD belonged to the PLD superfamily.

### 2.2. Recombinant Expression and Purification of Wild-Type MsPLD

The mature MsPLD was heterologously expressed in *E. coli* Shuffle T7. The recombination expression conditions of MsPLD were optimized, with IPTG of 0.2 mM, an induction temperature of 16 °C, along with a 20 h incubation, which were determined to be the ideal conditions to acquire recombinant MsPLD. With the help of Ni^2+^ affinity chromatography, the recombinant MsPLD was purified (Figure S2A). An SDS-PAGE assessment indicated that the recombinant MsPLD had a molecular weight of about 62 kDa, which was quite close to the theoretical molecular weight. Supplementary Table S1 lists the protein contents along with the catalytic activity recovered throughout the purification procedure. From 10 g wet cell weight, approximately 250 mg of pure MsPLD was acquired, and the specific activity of pure MsPLD was 13.4 U/mg with L-*α*-phosphatidylcholine (PC) from soybean as a substrate.

### 2.3. Enzyme Characterization of MsPLD

#### 2.3.1. Optimum Temperature and Thermostability of MsPLD

**Figure 1:**
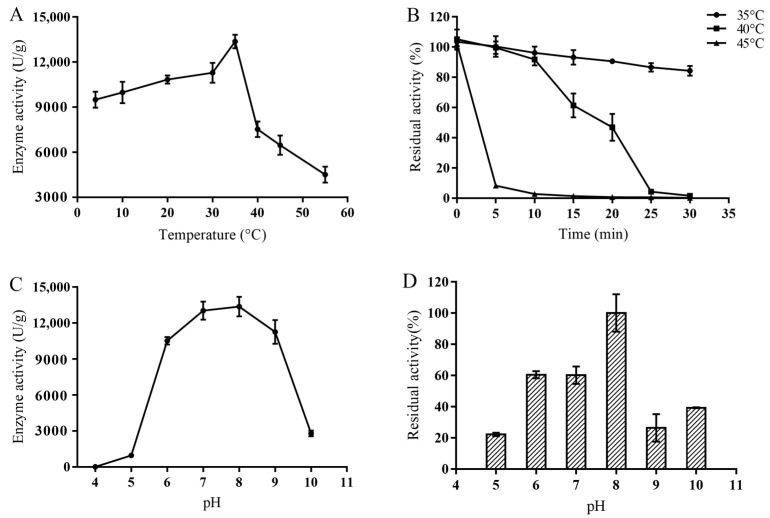
Enzymatic characterization of recombinant wild-type MsPLD. (**A**) Effects of temperature on enzyme activity. (**B**) Thermal stability of MsPLD under various temperatures. (**C**) Effects of different pH on enzyme activity. (**D**) pH stability of MsPLD. Results are expressed as mean ± SD (bars) of three independent experiments. The residual activity was calculated by defining the highest activity of samples as 100%.

The PLD showed the highest enzymatic activity at 35 °C, which was 13.37 ± 0.45 U/mg. Surprisingly, the enzyme still exhibited a certain enzyme activity at 4 °C, which was 70.99% of its maximum (Figure 1A). However, it showed poor thermal stability, and the half-life at 35 °C was only 117 min. When the MsPLD was treated at 40 °C and 45 °C for 25 min, the residual enzyme activity dropped below 20% (Figure 1B). MsPLD was very stable at 4 °C, and even after 18 days, the residual enzyme activity of the MsPLD was still above 70%. According to the enzyme inactivation curve, the half-life of the enzyme at 4 °C was determined to be 41 days.

#### 2.3.2. Optimum pH and pH Stability of the MsPLD

The optimal reaction pH of PLD was 8.0, and under the acidic conditions of pH 4.0–5.0, basically no activity was observed. The enzyme activity of the MsPLD rapidly decreased to about 20% of the optimal enzyme activity at alkaline condition of pH 10.0 (Figure 1C). The residue activity of the enzyme at pH 6.0–8.0 at 4 °C for 12 h was above 60%, indicating a good stability in the neutral conditions (Figure 1D).

#### 2.3.3. Metal Ion, Organic Solvent, and Surfactant Stability of the MsPLD

**Figure 2:**
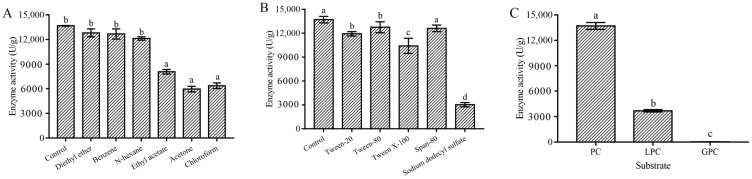
Enzymatic activities of wild-type MsPLD under different organic solvents (**A**), different surfactants (**B**), and different phospholipid substrates (**C**). Values are expressed as mean ± SD (bars) of three independent experiments. Different letters indicate that there was a significant difference between different groups (*p* < 0.05).

Results indicated that Mn^2+^ and Ca^2+^ could promote the enzyme activity of MsPLD, however, Cu^2+^, Fe^2+^, Fe^3+^, Zn^2+^ and the high concentration of Ni^2+^ could significantly inhibit the activity of MsPLD (Table S2). MsPLD showed a notable tolerance to organic reagents such as *n*-hexane, diethyl ether, and benzene, and the residue activity under these organic solvents was more than 85%, which was significantly higher than that of other organic reagents (*p* < 0.05) (Figure 2A). Among various surfactants tested here, Span-80 showed basically no effect on enzymatic activity. There was no significant difference between Tween-20 and Tween-80 on enzyme activity (*p* > 0.05); however, a strong inhibition effect on enzyme activity was found in sodium dodecyl sulfate, and its residual activity was only below 20% (Figure 2B).

#### 2.3.4. Substrate Selectivity and Kinetic Parameters of MsPLD

No hydrolysis activity was found for L-*α*-glycerophosphorylcholine (GPC), while the enzyme activity of MsPLD for L-*α*-lysophosphatidylcholine (LPC) was only 27% of that of soy PC (Figure 2C). Under the optimal conditions, the kinetic parameters of MsPLD to soy PC were determined. The *K_m_*, *k_cat_*, *k_cat_/K_m_*, and V_max_ values were tested to be 3.44 ± 0.81 mM, 10.75 ± 0.09 s^−1^, 3172.18 ± 34.90 s^−1^·M^−1^, and 16.91 ± 0.17 nmol·min^−1^, respectively.

### 2.4. Crystallization and Structural Analysis of the MsPLD

The single-wavelength anomalous diffraction (SAD) approach was adopted to acquire the crystal structure of the apo-MsPLD at a 2.3 Å resolution. It was part of the P2_1_2_1_2_1_ space group, which had unit-cell characteristics of a = 93.90 Å, b = 216.50 Å, c = 277.45 Å, α = 90 Å, β = 90 Å, and γ = 90 Å. Six molecules were identified in a single asymmetric unit. One asymmetric unit had a total of 984 water molecules. The final model’s Rfactor and Rfree values were 18.05% and 20.18%, respectively (Table S3).

#### 2.4.1. Overall Structure

MsPLD showed a typical β-α-β-α-β sandwich fold in its overall structure (Figure S3A). MsPLD had 21 strands, 12 helices, and a sequence of organized loops that came together to create an asymmetric globular shape (Figure S3B,C). Two strongly interconnected HKD domains with somewhat distinct topologies made up the structure. With a structural superimposition of MsPLD (PDB ID: 7WU1) with PLDs of *Streptomyces* sp. PMF PLD (PDB ID: 1F0I) and SaPLD (PDB ID: 2ZE4), the two conserved HKD motifs were highly overlapped in spatial location with Histidine258 and Histidine498 as the catalytic residues (Figure S3D).

#### 2.4.2. An Extra Loop Segment Was Found in MsPLD Compared to Other Reported PLDs

**Figure 3:**
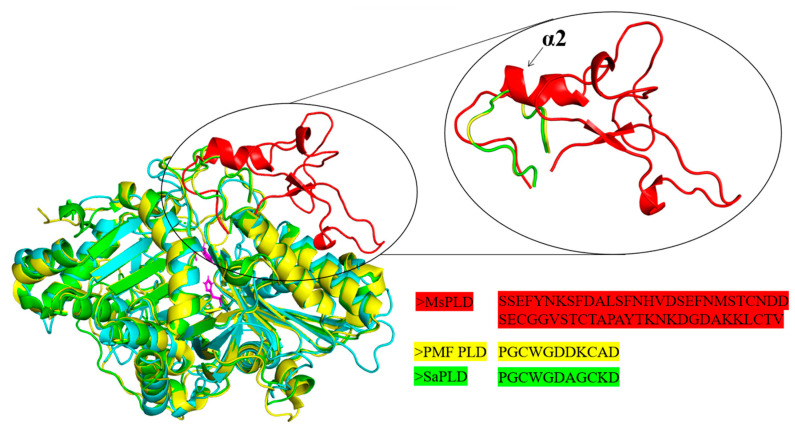
Superimposition and comparison of the structure of MsPLD (cyans, PDB ID: 7WU1) with other *Streptomyces* PLDs. *Streptomyces* sp. PMF PLD (yellow, PDB ID: 1F0I), SaPLD (green, PDB ID: 2ZE4). Two catalytic sites and the extra loop of MsPLD are shown in purple sticks and red color, respectively. The enlarged circle shows the unique extra loop motif that exists in MsPLD. The amino acids sequences that composed the corresponding loop are shown underneath.

With the superimposition and comparison of the structure of MsPLD (PDB ID: 7WU1) with other *Streptomyces* sp. PMF PLD (PDB ID: 1F0I) and SaPLD (PDB ID: 2ZE4), a unique extra loop motif that existed in MsPLD was found between β2 and α3. This extra loop was composed of one small helix (α2) and two sheets (β3 and β4) connected by a large loop. In contrast, a shorter loop was observed on *Streptomyces* sp. PMF PLD and SaPLD (Figure 3).

#### 2.4.3. Deletion or Fasting of the Extra Loop Segment Resulted in Complete Loss of Its Hydrolysis Activity

In order to clarify the function of the extra loop on the MsPLD, two truncation mutants (Del65-113, Del57-113) and one substitution mutant (MsPLD-1F0I) on the extra loop segment of MsPLD were designed based on the structural superimposition. The mutant protein was purified (Figure S2B). However, we found that the hydrolysis activity of two truncation mutants was completely lost. Moreover, the substitution of the corresponding segment of *Streptomyces* sp. PMF PLD (MsPLD-1F0I) also caused the complete loss of its activity to PC.

**Figure 4:**
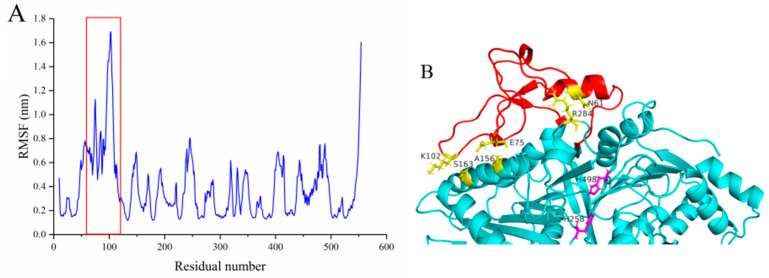
(**A**) RMSF plots for the MD simulations of wild-type MsPLD. Red box highlighted the RMSF value of the location of the extra loop of MsPLD. (**B**) Mutation sites selected for corresponding disulfide bonds construction are shown in yellow sticks. Two His258 and H498 located at the active site and the extra loop of MsPLD are shown in purple sticks and red color, respectively.

The exploration of the root-mean-square fluctuations (RMSFs) of MsPLD structures at temperature (473 K) illustrated that the corresponding extra loop segment (T55-P113) displayed high root-mean-square fluctuation (RMSF) values, which indicated that these regions were more flexible (Figure 4A). We thus constructed three extra disulfide bonds to verify whether the higher flexibility of this extra loop segment was required for the activity exhibition of MsPLD (Figure 4B). Three mutants were constructed and purified (Figure S2C). An enzyme activity assay was conducted, and the results showed that fastening the loop also caused the loss of the activity of MsPLD.

#### 2.4.4. Deletion of the Extra Loop Segment Significantly Decreased the Binding Properties of MsPLD to PC Monolayer

**Figure 5:**
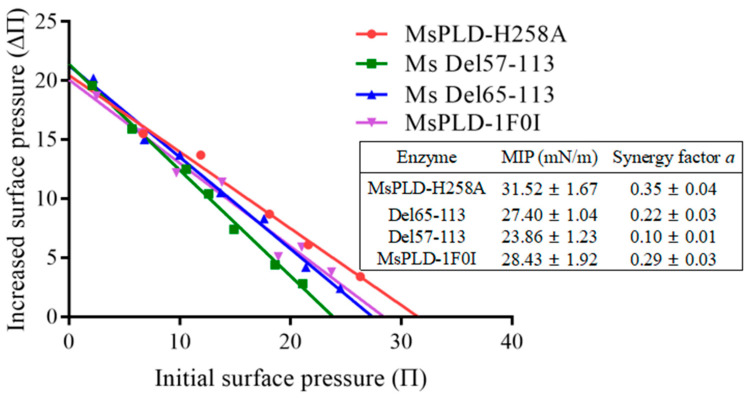
Interaction of wild-type MsPLD and its mutants with soy-PC monolayers. Maximal increase in surface pressure after injection of wild-type MsPLD or its mutants as a function of soy-PC monomolecular films that spread at various initial surface pressures. Maximum insertion pressure (MIP) and synergy factor a of wild type MsPLD and its mutants are shown in the table.

As an interfacially reactive enzyme, PLD acts at the lipid–water interface. Interfacial adsorption is the first step during the catalytic process. The binding of the enzyme to the lipid–water interface is thus considered to be a key step in controlling the substrate specificity and activity [24]. Based on the above research results, we wanted to further clarify whether the extra loop segment could affect the interfacial binding capacity of MsPLD and its mutants to the lipid–water interface. In order to study separately the absorption step of wild-type MsPLD and its mutants on soy-PC monolayers, a mutation H258A on the catalytic residue His258 was thus introduced to MsPLD and its mutants to remove their catalytic functions. The corresponding His258Ala mutant based on wild-type and various mutants was further constructed for studying such phospholipid–protein interactions independently from the substrate hydrolysis. A high purity of inactive wild-type MsPLD (MsPLD-H258A) and its corresponding truncation mutants were obtained (Figure S2C). The effects of different initial surface pressure (Π_i_) on the adsorption of MsPLD-WT and its mutants onto PC monolayers are shown in Figure S4. With the increase of time, the enzyme adsorption process showed an increasing trend at first and then it stabilized. The curve approaching equilibrium indicated that the enzyme adsorption process had reached a dynamic equilibration. When plotting ΔΠ_max_ as a function of Π_i_, a linear regression was obtained and its intercept to the x-axis allowed the determination of the MIP, and the synergy factor “*a*” was obtained as the slope +1. The MIP for the protein penetration was then defined: it corresponded to the extrapolated Π_i_ beyond which no increase in the surface pressure occurred. As shown in Figure 5, for the MsPLD-H258A, the MIP value was 31.52 ± 1.67 mN/m. However, two truncation mutants (Del65-113 and Del57-113) decreased to 27.40 ± 1.04 mN/m and 23.86 ± 1.23 mN/m, respectively. The MIP of the substitution mutant (MsPLD-1F0I) also significantly decreased to 28.43 ± 1.92 mN/m (*p* < 0.05).

The synergy factor *a* is another parameter employed to highlight the favorable binding of protein toward the monolayer film. It has been shown that such a positive synergy reveals a favorable binding. We found that all the proteins investigated here had a synergy factor *a* > 0 (Figure 5), indicating that a positive interaction occurred between proteins and substrates. However, compared to the wild-type MsPLD, a significant decrease in the value was also observed, which was consistent with the results of the MIP.

### 2.5. Application of MsPLD for the Synthesis of PA by Using PC as Substrate

**Figure 6:**
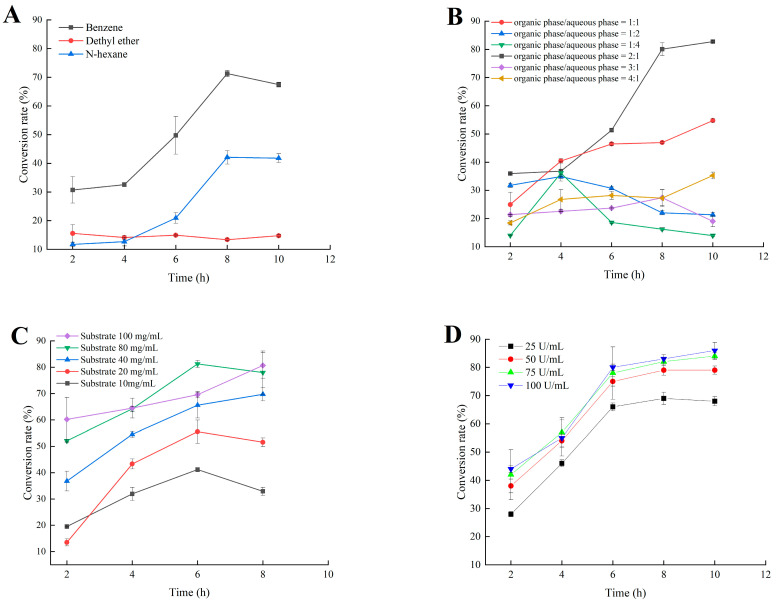
Effect of various organic solvents (**A**), benzene to aqueous phase ratio (**B**), substrate concentration (**C**), and enzyme amount (**D**) on the conversion rate of PA by using soy-PC as substrate.

In this enzymatic reaction, four parameters, including the organic solvent, volume ratio between the organic phase and the aqueous phase, the PC substrate concentration, and enzyme loading might have potential impacts on the PA conversion. Therefore, the effects of these parameters on the reaction were investigated to maximize PA conversion (Figure 6).

Combined with the above experimental results on the organic solvent tolerance of MsPLD (Figure 2A), three organic solvents were used in the present study to construct the biphasic reaction system, with benzene having the highest conversion rate, followed by ethyl ether, and the lowest conversion rate was observed while using *n*-hexane (less than 15%) (Figure 6A). When comparing various benzene and aqueous phase volume ratios, a substantially higher conversion rate was attained with the increase in the ratio of benzene. The highest conversion rate was found at 2:1. Further increasing the benzene ratio caused a significant reduction in the conversion rate (Figure 6B).

With the increase in the PC substrate, the conversion rate increased as well. The highest conversion rate was found at 80 mg/mL in the reaction system. Further, increasing the PC concentration to 100 mg/mL had no significant enhancement on the conversion rate (Figure 6C). Finally, the effect of enzyme loading on the conversion rate of PA was investigated. The conversion rate increased with the loading of enzyme. No significant difference in the conversion rate between 75 U/mL and 100 U/mL enzyme loading was found (*p* > 0.05) (Figure 6D). When continuing to increase the PC concentration and enzyme loading the conversion rate no longer increased, suggesting that in addition to the substrate and enzyme loading, the mass transfer may be critical for improving the productivity of MsPLD [25]. Combining all the above results, we finally determined that the best reaction system and condition used for the synthesis of PA were benzene: aqueous 2:1 (*v*/*v*), 80 mg/mL PC, 75 U/mL enzyme loading, and reaction at 35 °C and 800 rpm. Under these conditions, the conversion rate of PA reached 86% after a 10 h reaction. Moreover, the PA production lasted for 10 h indicating that the enzyme was more stable in the presence of the substrate compared to exsit alone at 35 °C [25].

## 3. Discussion

**Figure 7:**
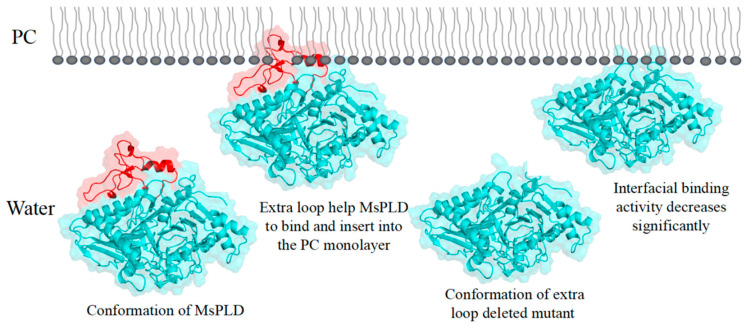
Schematic model of interfacial binding of wild type MsPLD and its truncated mutant to the PC monolayer. Proposed anchor role of extra loop segment on MsPLD binding to the PC monolayer. The extra loop of MsPLD is marked in red.

Despite the growing number of PLDs found at the genetic level in many organisms and multiple reports on a PLD-mediated synthesis of tailor-made phospholipids with functional head groups, information on the structural–functional relationship remains scare [26]. Obtaining structural information on PLDs is crucial for understanding the catalytic mechanisms of enzymes as well as providing guidelines for enzyme engineering. In this study, we determined the crystal structure of MsPLD for the first time. An extended unique loop in MsPLD was noticed. Dynamic simulation results indicated that the loop segment was flexible during the simulation. Results indicated that direct deletion, fastening, or substitution of the extra loop segment all resulted in a complete loss of its activity. These results suggested that it may play a certain role during enzyme activity exhibition. Since the detail function of the extra loop segment of the MsPLD had not been determined, in order to explain the possible causes of its loss of activity for these mutants, we performed an in-depth analysis of the partial superposition structure of MsPLD to *Streptomyces* sp. PMF PLD (PDB ID: 1F0I) and SaPLD (PDB ID: 2ZE4) (Figure S5). We noticed that there were two other loops near our extra loop segment, which were considered to play an important role on the substrate entrance to the active site and the recognition of phospholipids on *Streptomyces* PLDs [16]. Moreover, the results of a polar interaction analysis showed that two hydrogen bonds were formed between residues of R283 and D64, E491 and S55, and S56 between these loops, indicating that except the two previously reported loops, the extra loop segment found in MsPLD may also participate in phospholipid recognition and help the entrance to the catalytic pocket of MsPLD (Figure S5). Either truncating or replacing this extra loop segment would result in the loss of its interaction with the other two loops, leading to a change of the local structure and further affecting the formation of the substrate entrance and the recognition of phospholipid substrates, and ultimately leading to the loss of MsPLD’s enzymatic activity. Further research is still needed to clarify the detailed mechanism. Furthermore, fastening the loop to the enzyme also resulted in the loss of enzyme activity; this experimental result further verified our dynamic simulation result and suggested that maintaining a flexible state of this extra loop segment was also very important for MsPLD’s enzyme activity exhibition. In addition, considering that the PLD are generally typical of lipid-converting enzymes, PLDs need to bound to the phospholipid interface to gain access to their substrates. Moreover, interfaces are also critical for the complete functionality of PLDs [27]. Thus, the interfacial binding characteristics of wild-type MsPLD and its truncation mutants were examined. The additional loop segments of MsPLD played a key role in interfacial binding and insertion into phospholipid monolayers, according to the MIP and synergy *a* analysis results. Removing the loop section would reduce the protein’s contact with the monolayer, lowering its binding characteristics. A topological model of MsPLD binding to the interface was proposed based on these findings (Figure 7). The additional loop segment is flexible under aqueous solution. When phospholipids substrates are present, MsPLD travels to the lipid–water interface, where a portion of the additional loop segment is inserted into the phospholipid monolayers, allowing the substrate to migrate into the catalytic pocket and undergo catalytic reaction. MsPLD could still maintain its capacity to bind to phospholipids after the additional loop segment has been removed from its structure. However, unlike wild-type MsPLD, the truncated mutant protein could not penetrate deeply enough into the membrane interface to allow for a more stable binding. The lower insertion and binding capacity of the truncated mutant protein may further affect its enzymatic activity (Figure 7).

PLDs are now recognized as an important tool for the enzymatic synthesis of PLs. The important application values of PLDs have been expected for a long time. Previous studies on PLDs mainly focused on their transphosphatidylation activity to synthesize naturally rare and novel artificial phospholipids (PLs) from highly abundant phosphatidylcholines (PCs) through transphosphatidylation reactions [26]. During this reaction, PA acted as a by-product and was usually not desirable. As we mentioned in our studies, PA has important application values in different industries. Considering this situation and the much higher price of PA compared to PC, the enzymatic synthesis of PA is still relevant. Pasker investigated the influence of enzymatic extracts from savoy cabbage (PLDsc), white cabbage (PLDwc), and brussels sprouts (PLDbs) on rapeseed phosphatidylcholine (RPC) to prepare rapeseed phosphatidic acid (RPA) [28]. A hydrolysis reaction was carried out in the biphasic system (water/diethyl ether) and reaction conditions were optimized. The highest activity towards RPC showed a PLD extract from PLDsc with a RPC conversion degree to RPA of 90%. In our present work, biphasic systems were also selected to evaluate the influence of various parameters on the conversion rate. Compared to the aqueous system, the conversion ratio in a biphasic reaction system was significantly improved. These results indicated that the lower solubility of PC in the aqueous system may not facilitate the reaction of PC with enzyme. Similar results were also found during the transphosphatidylation reactions to produce PS or other PLs, further demonstrating the importance of interface on the reaction [16]. In spite of this, considering that the organic solvent used in the present reaction has the residual risk and is much more toxic, further studies are still needed to screen and find other green reaction systems, for example, using low toxicity organic solvents or deep eutectic solvents.

## 4. Materials and Methods

### 4.1. Chemicals and Reagents

L-*α*-phosphatidylcholine from soybean [14], L-*α*-lysophosphatidylcholine (LPC), L-*α*-glycerophosphorylcholine (GPC), L-*α*-phosphatidic acid (PA) were all purchased from Sigma-Aldrich (St. Louis, MO, USA). Choline oxidase used in the enzyme assay was prepared by the previously reported method [29]. Horseradish peroxidase, isopropyl-*β*-D-1-thiogalactopyranoside, ampicillin, and the Bradford Protein Assay Kit were purchased from Sangon Biotech Co., Ltd. (Shanghai, China). *Escherichia coli* Top10 was used as the host for the construction and proliferation of plasmid DNA, and *Escherichia coli* SHuffle T7 was used as the host for the expression of wild-type MsPLD and its mutants. Its corresponding competent cell was purchased from New England BioLabs (Beijing, China). Plasmid pET-21a (Novagen, Darmstadt, Germany) was used for the construction of the expression vector. Ni^2+^-nitrilotriacetate (Ni^2+^-NTA) affinity column, desalting column, and Hiload 16/60 Superdex 200 pg gel filtration column were obtained from GE Healthcare Life Sciences (Pittsburgh, PA, USA). PrimeSTAR HS DNA polymerase, restriction endonucleases, PCR reagents were purchased from TaKaRa (Dalian, China). The eleven crystallization kits (crystal screen 1 and 2 (HR2-130), Wizard Class 1 and 2 and 3 and 4, SaltRx (HR2-107, HR2-109, HR2-136), Index (HR2-134) and Stock Options pH (HR2-241)) were all purchased from Hampton research company (Aliso Viejo, CA, USA). All other chemicals and reagents were of analytical grade or higher quality.

### 4.2. Bioinformatic Analysis of MsPLD

The MsPLD protein from *Moritella* sp. JT01 expressed in this study was deposited in the NCBI-Protein databases under the accession number KXO13223.1. The signal peptide of MsPLD was predicted using the online tools SignaIP v. 5.0 web server (http://www.cbs.dtu.dk/services/SignalP/) (accessed on 5 March 2021) [30]. Amino acid sequence alignment of various PLDs was analyzed by ClustalW (https://www.genome.jp/tools-bin/clustalw) (accessed on 5 March 2021) and imaged by ESPript 3.0 (http://espript.ibcp.fr/) (accessed on 5 March 2021) [31].

### 4.3. Recombinant Expression of MsPLD in Escherichia coli

The gene encoding the mature protein of MsPLD (without the signal peptides) was artificially synthesized by Sangon Biotech Co., Ltd. (Shanghai, China) and inserted into the pET-21a expression vector between the NdeI and XhoI restriction sites to yield the expression vector pET21a-MsPLD(His)_6_ (C-terminal with 6×His tag). Plasmids were confirmed by sequencing. The constructed vector was further transformed into an *E. coli* SHuffle T7 express competent cell. The recombinant strain was cultured at 37 °C in Luria–Bertani (LB) medium containing the corresponding antibiotics (0.1 mg/mL) and induced by 0.2 mM isopropyl *β*-D-1-thiogalactopyranoside (IPTG) at 16 °C for 20 h. Cells were harvested and resuspended in buffer A (50 mM Tris-HCl, 500 mM NaCl, pH 8.0), and disrupted by sonication on ice. The lysates were centrifuged at 4 °C, 8000 rpm for 30 min, and the supernatants were collected for further purification. The supernatant was loaded onto Ni^2+^-NTA packed columns and washed with buffer A plus 40 mM imidazole. The target proteins were eluted with buffer B (50 mM Tris-HCl, 500 mM NaCl, 200 mM Imidazole, pH 8.0) [26]. Fractions containing the proteins were then loaded onto the desalting column and washed with buffer A. Samples of the target proteins were analyzed by using 12% SDS-PAGE. The protein concentration was determined with the Bradford protein assay kit.

### 4.4. Enzyme Characterization

#### 4.4.1. MsPLD Hydrolysis Activity Assays

The hydrolysis activity of MsPLD was assayed using the enzyme-linked colorimetry method described by Shimbo et al. with minor modifications [32]. An enzymatic assay mixture (100 μL) containing 0.1 M Tris-HCl (pH 8.0), 5 mM PC, 15 mM SDS, 15 mM Triton X-100, and purified enzyme solution was incubated at 35 °C for 10 min, the enzyme reaction was terminated by heating at 100 °C for 5 min, the solution was cooled, and a 50 μL mixture of 10 U/mL choline oxidase, 1 U/mL peroxidase, 5 mM 4-aminoantioyrine, and 7 mM phenol were added and further incubated at 30 °C for 60 min. The absorbance was tested at 500 nm. One unit of enzyme activity was defined as the amount of enzyme required to release 1 μmol choline per minute under the assay conditions. Experiments were performed in triplicate.

#### 4.4.2. Effects of Temperature and pH on Enzyme Activity

Enzymatic assays were performed under different temperatures (4–55 °C) and different pH values (4.0–10.0) to determine the optimum reaction condition of MsPLD by using PC as a substrate. Various buffers used in this study included 50 mM citric acid-sodium citrate buffer (pH 4.0 and 5.0), 50 mM phosphate buffer (pH 6.0 and 7.0), 50 mM Tris-HCl (pH 8.0), and 50 mM Gly-NaOH (pH 9.0 and 10.0). The thermal stability was tested by preincubating the MsPLD at different temperatures (4 °C, 35 °C, 40 °C, 45 °C). Samples were then taken at different intervals for the measurement of residual activity under the optimal reaction conditions. The highest activity was designated as 100%. The relative activity at other temperatures was obtained through a comparison with the highest activity for the same enzyme. The pH stability of MsPLD was determined by preincubating the enzyme in different pH buffers (4.0–10.0) at 4 °C for 12 h. The residual activity was then determined under the optimal reaction conditions. The highest residual activity at the corresponding pH was designated as 100%. To further evaluate its storage stability, MsPLD was stored at 4 °C and pH 8.0 and the residual enzymatic activity was detected at an interval of two days. The half-life of MsPLD under different temperatures was calculated.

#### 4.4.3. Metal Ion, Organic Solvent, and Surfactant Stability of MsPLD

To evaluate the effects of various metal ion on the PLD hydrolysis activity, the enzyme was preincubated in 5 mM and 10 mM final concentrations of various metal ions (Cu^2+^, Ni^2+^, Mn^2+^, Ca^2+^, Fe^2+^, Fe^3+^, Mg^2+^, Zn^2+^) at 4 °C for 1 h, respectively. Then, enzyme-linked colorimetry was used to determine the hydrolysis activity of the MsPLD by using PC as substrate. The effects of various organic solvents (ethyl acetate, *n*-hexane, benzene, diethyl ether, chloroform, and acetone) on MsPLD activity were determined. The enzyme solution was incubated separately with each organic solvent (final concentration 50%, *v*/*v*) at 4 °C for 1 h, and then the residual activity of the enzyme was measured at 35 °C and pH 8.0 by using PC as a substrate. The effects of various surfactants (SDS, Triton X-100, Tween-20, Tween-80, Span-80) on MsPLD activity were determined. The enzyme solution was incubated separately with each surfactant (final concentration 1% (*v*/*v*)) at 4 °C for 2 h and then the residual activity of the enzyme was measured at 35 °C and pH 8.0 by using PC as substrate. The enzyme activity without reagent treatment was designated as 100%.

#### 4.4.4. Substrate Selectivity and Kinetic Parameters

The substrate selectivity of MsPLD to various glycerol phosphatidylcholine (PC, LPC, GPC) was measured at 35 °C and pH 8.0. The kinetic parameters of MsPLD to PC were further determined by using enzyme-linked colorimetry with different substrate concentrations (0.5–8.0 mM). The apparent affinity constant [33] and turnover number (*k_cat_*) values of the reaction were obtained by using the Michaelis–Menten kinetic equation.

### 4.5. Crystallization and Structure Determination

The preliminarily Ni^2+^-NTA affinity-column-purified protein was further purified by a Hiload 16/60 Superdex column which was obtained from GE Healthcare Life Science (Pittsburgh, PA, USA) and used for protein crystallization [34]. A preliminary screening for the crystallization conditions of MsPLD was carried out using the hanging drop technique at 16 °C. Eleven crystallization kits were used to screen the best growth conditions of MsPLD crystal. A series of two factor variable orthogonal experiments were performed to optimize the precipitant concentration and the pH of the crystallization reagent. Eventually, a high-quality monocrystal of MsPLD was obtained under the condition of 20% PEG 1000, 0.1 M disodium hydrogen phosphate/citric acid pH 4.2, and 0.2 M lithium sulfate. Crystals were rapidly immersed in reservoir solution with 20% glycerol as a cryoprotectant, and then flash-cooled in nitrogen flow. Diffraction images were collected at beam line 18U1 of the Shanghai Synchrotron Radiation Facility (SSRF, Shanghai, China). Diffraction data were processed using the XDS package [8]. Manual model building was performed with COOT [35] and the crystal structure of the MsPLD apo-state structure was directly solved by the molecular replacement (MR) method by the program PHASER in the CCP4 program suite [36]. Crystallographic refinements were executed using PHENIX, and the qualities of the final model were evaluated by PROCHECK [37,38]. Finally, the coordinates and structural factors of MsPLD were deposited in the Protein Data Bank (PDB) with PDB ID: 7WU1. Diagrams of the protein structure were drawn by PyMOL software (version 2.6, created by Warren L. DeLano) [39].

### 4.6. Molecular Dynamic Simulations of MsPLD

Molecular dynamic (MD) simulations of wild-type MsPLD were performed using Gromacs 2019.6 with an Amberff99SB-NMR-ildn force field. The protein was solved in a cube box with TIP3P water, and the distance between the enzyme surface and the box boundary was at least 1.2 nm. Sufficient sodium ions were added for neutralizing the charges of the system. After minimizing the system energy, the temperature was slowly raised from 0 K to the 473 K for 500 ps. Then, a 50 ns production of MD of simulation was performed with a 2 fs time step in the NpT ensemble at 473 K. Analyses of the MD simulations were performed with Gromacs tools.

### 4.7. Structure-Based Mutants Design, Construction, and Expression

Two truncation mutagenesis (Del65-113, Del57-113) and one substitution mutagenesis (MsPLD-1F0I) on the extra loop segment of MsPLD were designed based on the structural superimposition and comparison with other PLDs to characterize the function of the extra loop segment on the activity and stability of MsPLD. Various mutageneses was carried out by the overlap extension method with the constructed pET21a-MsPLD-(His)_6_ plasmid as templates. Moreover, to ensure that the MsPLD had no hydrolysis activities toward the phospholipid monolayers, a single-site mutation in its catalytic active site (H258A) of MsPLD was further introduced and used for the following interfacial binding assay. The final products were digested with *Dpn* I and used to transform *E. coli*’s top 10 competent cells. These mutants were sequenced further to ensure their sequence accuracy before being transformed into *E. coli* SHuffle T7 for protein expression. The oligonucleotides used for mutagenesis are listed in Supplementary Table S4 in the Supplementary Materials.

### 4.8. Interfacial Adsorption Measurements by Using Monolayer Technology

Interfacial binding assays were performed by using Microtrough from Kibron (Helsinki, Finland) according to the methods reported before [27]. The inactive form of wild-type MsPLD and its various mutants’ adsorption onto PC monolayers was observed until its corresponding equilibrium surface pressure (Πe) was reached. The values of the maximum insertion pressure (MIP) and synergy factor ‘*a’* parameters were calculated and used to characterize interfacial binding properties of various enzymes to PC monolayer according to the method reported before [15].

### 4.9. Enzymatic Synthesis of PA by MsPLD

#### 4.9.1. Reaction System Optimization

A biphasic reaction system was used for the enzymatic synthesis of PA by MsPLD. The basic reaction conditions were as follows: a reaction mixture consisting of PC was dissolved in organic phase as a substrate, and purified MsPLD was added to the buffer solution (50 mM Tris-HCl buffer, pH 8.0) to catalyze the synthesis of PA. The reaction conditions of various organic phases (benzene, diethyl ether, and *n*-hexane), the proportion of organic phase to aqueous phase (1:1, 1:2, 1:4, 2:1, 3:1, and 4:1 *v*/*v*), the PC substrate concentration (10 mg/mL, 20 mg/mL, 40 mg/mL, 80 mg/mL, and 100 mg/mL), and enzyme loading (25 U/mL, 50 U/mL, 75 U/mL, and 100 U/mL) were varied for optimization. Reactions were carried out in a water bath at 35 °C, 800 rpm. Samples were taken every two hours and mixed with 25 µL of 1 mol/L HCl to inactivate the enzyme. Phospholipids were extracted with equal volume of chloroform/methanol (2:1, *v*/*v*) and analyzed by high-performance liquid chromatography. The PA conversion rate [32] was defined as the percentage of PA obtained compared with the initial concentration of PC.

#### 4.9.2. Analysis of PA by High-Performance Liquid Chromatography

PA in the reaction mixture was analyzed via high-performance liquid chromatography equipped with an evaporation light detector. The column was a Hyperil GOLD silica (250 mm × 4.6 mm × 5 μm) column, which was maintained at 35 °C. Mobile phase A was composed of methyl alcohol/water/acetic acid (85:15:0.5, *v*/*v*/*v*) with 0.05% triethylamine (TEA), and mobile phase B was composed of *n*-hexane/2-propanol/mobile A (20:45:20, *v*/*v*/*v*). Elution conditions were as follows: 0–5 min, 60% A; 10 min, 90% A; 11 min, 0% A; and 14 min, 0%A. The flow rate was 1.0 mL/min, the evaporating temperature was controlled at 85 °C, and the flow rate of air gas was 2 L/min. Each phospholipid was determined from the retention time by using calibration solutions of phospholipids (PC, PA), and the concentrations of the phospholipids in the samples were calculated from the peak areas by integration.

### 4.10. Statistical Analysis

All experiments were performed in triplicate and results were given as mean ± SD. The significance of difference at *p* value of 0.05 was determined by using an ANOVA procedure.

## 5. Conclusions

In the present studies, a new PLD from *Moritella* sp. JT01 (MsPLD) was biochemically and structurally characterized for the first time. Furthermore, a preliminary exploration was carried out to characterize the function of the unique extra loop segment in the structure of this enzyme. Moreover, the application potential of MsPLD in the synthesis of PA based on its hydrolysis activity to a substrate of PC was evaluated and exhibited great application prospects. Although some explorations were conducted, more questions remain to be answered. For example, the mechanism of how the loop affects the substrate recognition and enzyme function of MsPLD remains elusive. In addition, the transphosphatidylation activity of MsPLD, its corresponding substrate spectrum, and the recognition mechanism deserve further study. Nevertheless, the present evaluation of MsPLD may help to gain an in depth understanding of the mechanism of MsPLD–phospholipid interactions and bring new insights into the rational design of novel PLDs with intriguing functions.

## Data Availability

Not applicable.

## Supplementary Materials

The following supporting information can be downloaded at: https://www.mdpi.com/article/10.3390/ijms231911633/s1.
