# Supplementary material for: A New Phospholipase D from Moritella sp. JT01: Biochemical Characterization, Crystallization and Application in the Synthesis of Phosphatidic Acid
Source: Int J Mol Sci. 2022 Oct 1;23(19):11633. doi: 10.3390/ijms231911633 (PMC9570413; doi:10.3390/ijms231911633)
Supplement: Supplementary file 1 [file ijms-23-11633-s001.zip › ijms-1818840-supplementary.pdf]

Supplementary Information

A new phospholipase D from *Moritella* sp. JT01: Biochemical characterization, crystallization and application in the synthesis of phosphatidic acid

Fanghua Wang [1], Xuejing Mao [1], Fuli Deng [1], Ruiguo Cui [1], Lilang Li [1], Siyu Liu [1], Bo Yang [2], Dongming Lan [1,*], Yonghua Wang [1,*]

[1] School of Food Science and Engineering, South China University of Technology, Guangzhou 510640, China

[2] School of Bioscience and Bioengineering, South China University of Technology, Guangzhou 510006, China

[*]Correspondence: dmlan@scut.edu.cn (D.L.); yonghw@scut.edu.cn (Y.W.), Tel.: +86-20-8711-3842

Supplementary Table S1 Summary on purification steps of wild type MsPLD.

Purification steps	Total activity (U)[a]	Total protein (mg)	Specific activity (U/mg)	Purification (fold)	Yield (%)
Crude extract	4111	1242.9	3.31	1.00	100.00
Ni^{2+}-NTA	3311	261.8	12.65	3.82	80.54
Desalting column	2682	200.6	13.37	4.04	65.23

[a] The specific activity and protein concentration were determined as described in methods section.

Supplementary Table S2 Effects of different metal ions on the activity of MsPLD.

Metal ion	Concentration (mM)	Relative activity (%)
None	0	100±0.26
Mn^{2+}	5	106.26±0.22
	10	109.99±0.85
Ca^{2+}	5	100.09±0.35
	10	104.12±1.93
Mg^{2+}	5	96.01±2.93
	10	96.50±1.05
Ni^{2+}	5	106.18±0.13
	10	99.28±6.16
Zn^{2+}	5	62.37±0.74
	10	66.40±2.85
Fe^{2+}	5	79.54±0.48
	10	7.14±4.64
Fe^{3+}	5	10.91±0.96
	10	3.83±2.83
Cu^{2+}	5	16.95±0.70
	10	8.94±1.10

Note: Enzyme activity without metal ions adding was defined as 100%.

Supplementary Table S3 Data collection and refinement statistics of MsPLD.

	MsPLD
Wavelength	0.97919
Resolution range	23.36-2.3 (2.38- 2.3)
Space group	$P2_12_12_1$
Unit cell	a =93.90 Å, b =216.50Å, c=277.45Å<br>α = 90Å, β =90Å, γ = 90Å
Unique reflections	250032 (24724)
Multiplicity	12.3(6.8)
Completeness (%)	99.69 (99.38)
Mean I/sigma(I)	0.6
Wilson B-factor	49.48
R-meas	0.178 (2.892)
R-pim	0.050 (0.889)
CC1/2	0.995 (0.304)
Reflections used in refinement	249785 (24611)
Reflections used for R-free	1742 (171)
R-work	0.1805 (0.2957)
R-free	0.2018 (0.3025)
Number of non-hydrogen atoms	26491
macromolecules	25452
ligands	55
solvent	984
Protein residues	3254
RMS(bonds)	0.010
RMS(angles)	0.94
Ramachandran favored (%)	93.72
Ramachandran allowed (%)	5.50
Ramachandran outliers (%)	0.77
Rotamer outliers (%)	1.01
Clashscore	5.30
Average B-factor	61.54
macromolecules	61.82
ligands	57.29
solvent	54.48
Number of TLS groups	25

Supplementary Table S4 Primers used in this study.

Primer	Sequence (5'to3')*
N61C-F	CACTTCCAGCGAATTTTACTGCAAATCCTTCGACGCGCTG
N61C-R	CAGCGCGTCGAAGGATTTGCAGTAAAATTCGCTGGAAGTG
E75C-F	GAGCTTCAACCACGTTGACTCTTGCTTCAACATGTCTACCTGTAACG
E75C-R	CGTTACAGGTAGACATGTTGAAGCAAGAGTCAACGTGGTTGAAGCTC
K102C-F	CACAGCACCGGCGTACACCAAAAACTGCGATGGTGATGCTAAAAAAC
K102C-R	GTTTTTTAGCATCACCATCGCAGTTTTTGGTGTACGCCGGTGCTGTG
A156C-F	TTTCTAGCGGTGCTTTCACCTGCACCATTAAAAACGCGCTGAG
A156C-R	CTCAGCGCGTTTTTAATGGTGCAGGTGAAAGCACCGCTAGAAA
S163C-F	CCATTAAAAACGCGCTGTGCCAGCTGGCGAAAAAC
S163C-R	GTTTTTCGCCAGCTGGCACAGCGCGTTTTTAATGG
R284C-F	GCGGATTATCTGCAGTGTAACCCGGTGAACG
R284C-R	CGTTCACCGGGTTACACTGCAGATAATCCGC
H258A-F	TGAACGTTGCTTGGAACGCCGGTAAAATTATCAACGTTGACAACC
H258A-R	TTTACCGGCGTTCCAAGCAACGTTCAGCAGAACATCTTTCTGGCTGTT
MsPLD-1F0I-1-F	CCCACTTCACCTTCGGCACTCCGGGTTGCTGGGGTGACGATGGTGATGCTAAAAAACT
MsPLD-1F0I-1-R	AGTTTTTTAGCATCACCATCGTCACCCCAGCAACCCGGAGTGCCGAAGGTGAAGTGGG
MsPLD-1F0I-2-F	ACTCCGGGTTGCTGGGGTGACGACAAATGCGCTGACGATGGTGATGCTAAAAAACTG
MsPLD-1F0I-2-R	CAGTTTTTTAGCATCACCATCGTCAGCGCATTTGTCGTCACCCCAGCAACCCGGAGT
Del57-113-F	GGATACCCACTTCACCTTCGGCACTCCGGCTGATAAAATTCTGGATGCGA
Del57-113-R	TCGCATCCAGAATTTTATCAGCCGGAGTGCCGAAGGTGAAGTGGGTATCC
Del65-113-F	TTCCAGCGAATTTTACAACAAATCCCCGGCTGATAAAATTCTGGATGCGA
Del65-113-R	TCGCATCCAGAATTTTATCAGCCGGGGATTTGTTGTAAAATTCGCTGGAA

* Underlined sequences were miss-match regions for site directed mutagenesis.

A

```
        10         20         30         40         50         60
MKIQNKHAMI ILKSAASAFV LVSASASAST NELDVNDIYD HLNEKYSQFN DVTFSKPSTN

        70         80         90        100        110        120
YLKPGWILDT HFTFGTSSEF YNKSFDALSF NHVDSEFNMS TCNDDSECGG VSTCTAPAYT

       130        140        150        160        170        180
KNKDGDAKKL CTVPADKILD AIYDNIVSAK RSVDIVTLQP MDISHLNLSF SSGAFTATIK

       190        200        210        220        230        240
NALSQLAKNT QYSDHHITVR LLQGSFTPML GYDAESEEEE IRQLSLTQTN YLSEIASVLP

       250        260        270        280        290        300
EVNNLDITVG SVRSCNKLIS NCGNNNSQKD VLLNVAWNHG KIINVDNQSV ITGGHNLWGA

       310        320        330        340        350        360
DYLQRNPVND LSINILGPIA STATKYGNTL WNYVCNNTGT ITNTFVTYAN GQYTYDCPAH

       370        380        390        400        410        420
ISSTYVAPTD AKNGLAVKVM SISKLNNGVL DKDADQSEVA RVYAFKNATK SIKISQQALF

       430        440        450        460        470        480
FKGAFGKVLH PLKTIDGTVM EALASAIYKG VTVDIVTSSL DGGIYSSGYN SEFVYNYLLN

       490        500        510        520        530        540
VLHKAPYYLE RNYAKTFLDK NLHINFISIN GRETNNMSHN KLWIVDDKVF YVGSHNIYPS

       550        560        570
SLQQFGVIVD DKDATAQLEK QLWTPMWKNS IHVPINNS
```

B

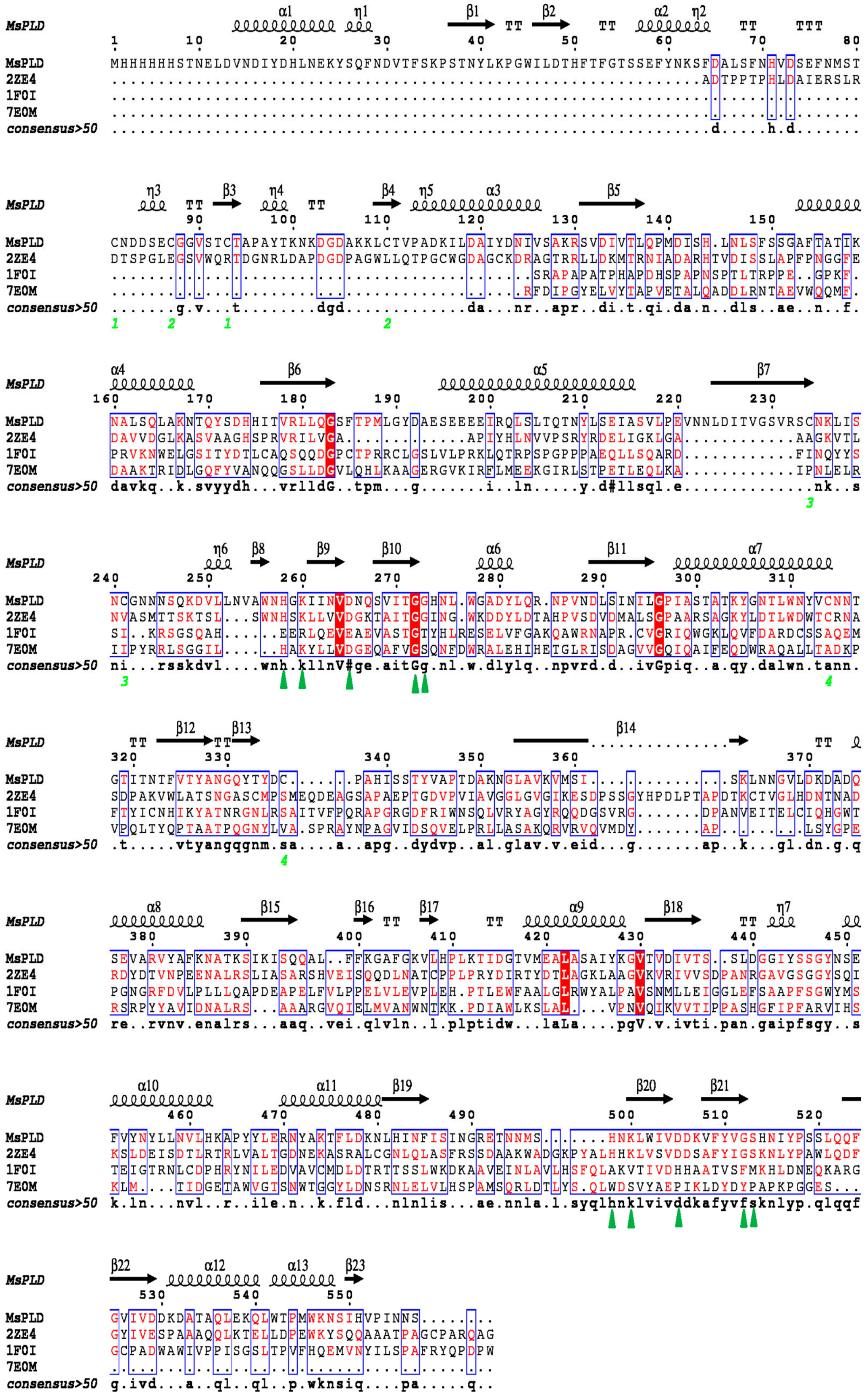

Supplementary Figure S1. Bioinformatic analysis of MsPLD. (A) Full length amino acid sequence of MsPLD. The deduced signal peptide that predicted with SignalP 5.0 server was labeled in purple. Two conversed HXK$(X)_4$D$(X)_6$GG/S (HKD) motifs of the PLD superfamily were shown in the red box and key residues were labeled in red. (B) Structural-based amino acid sequence alignment of the mature MsPLD with several other PLDs in the PDB. SpPLD (PDB ID: 7E0M), Streptomyces sp. PMF PLD (PDB ID: 1F0I), SaPLD (PDB ID: 2ZE4). Residues highlighted in blue box were indentical among the protein compared. Four pairs of disulfide bonds were numbered.

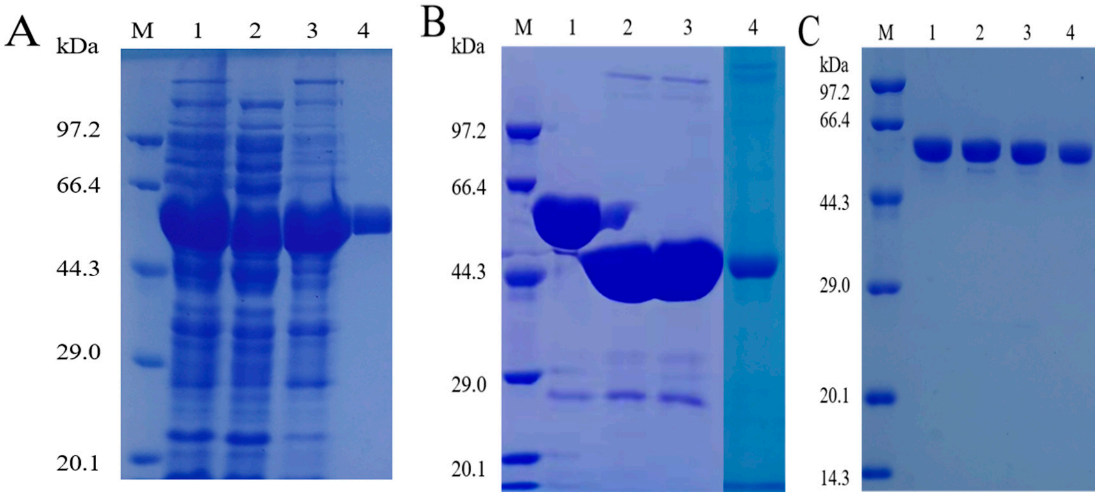

Supplementary Figure S2. Sodium dodecyl sulphate-polyacrylamide gel electrophoresis (SDS-PAGE) analysis of the recombinant wild type MsPLD and its corresponding mutants. (A) Wild type MsPLD. Lane M, The protein marker; Lane1, total cell extract; Lane 2, supernatant of total cell extract; Lane 3, pellet of total cell extract; Lane 4, purified MsPLD by using nickel column chromatography. (B) Purified protein of various mutants. Lane M, The protein marker; Lane 1, MsPLD-H258A; Lane 2, Del57-113; Lane 3, Del65-113; Lane 4, MsPLD-1F0I; (C) Purified protein of wild type and various mutants. Lane M, The protein marker; Lane 1, MsPLD; Lane 2, N61C-R284C; Lane 3, E75C-A156C; Lane 4, K102C-S163C.

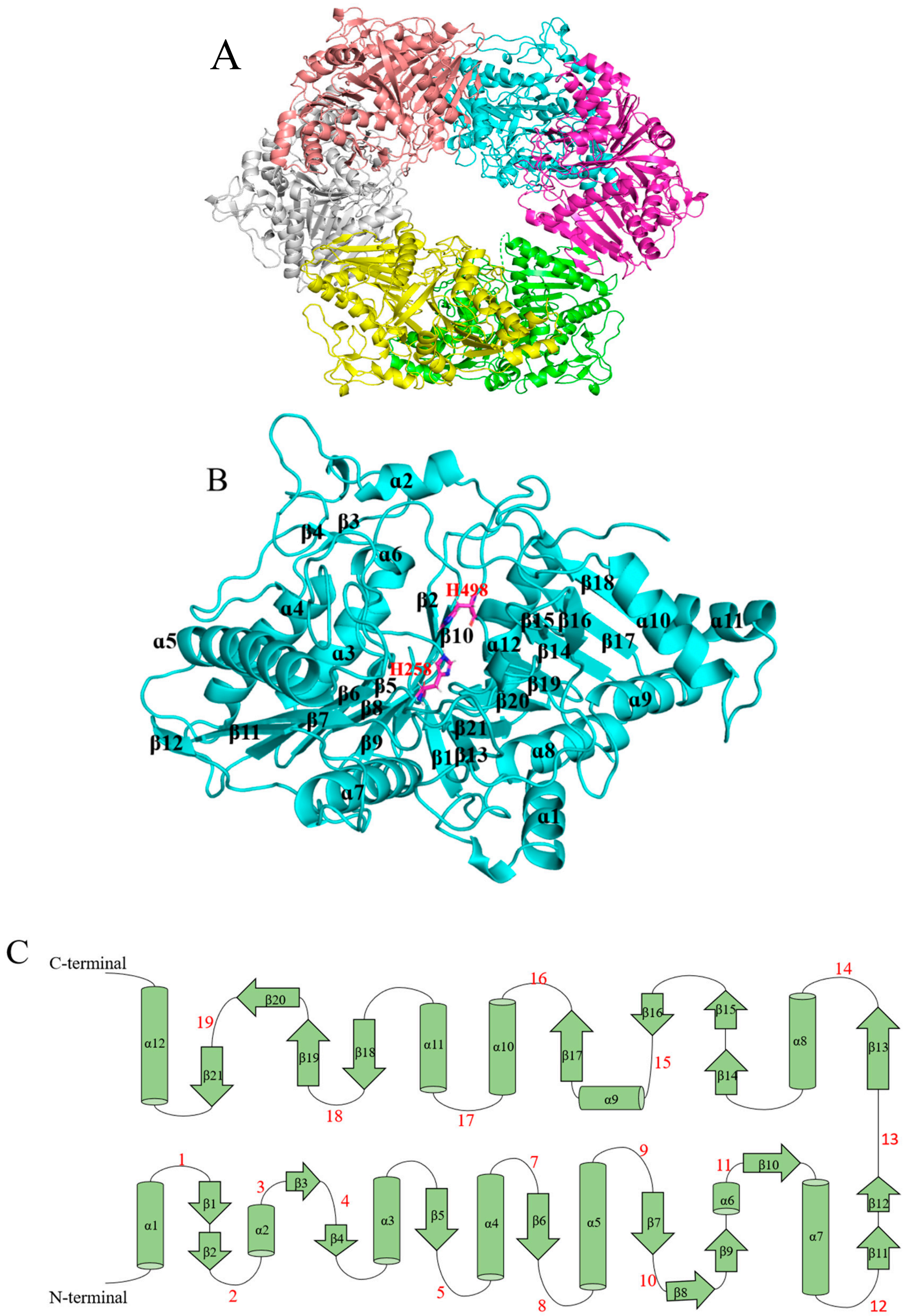

A
B
α2
β4 β3
α6
H498
β2
β18
α4
β15β16
α10
α11
α5
β10
α12
β17
β14
α3
H258
β5
β6
β8
β19
β20
α9
β7
β12
β11
β21
β9
β1β13
α8
α7
α1
C
C-terminal
N-terminal
α12 β21 β20 β19 β18 α11 α10 β17 α9 β16 β15 β14 α8 β13
α1 β1 β2 α2 β3 β4 α3 β5 α4 β6 α5 β7 β8 β9 α6 β10 α7 β11 β12
1 2 3 4 5 6 7 8 9 10 11 12 13 14 15 16 17 18 19

D

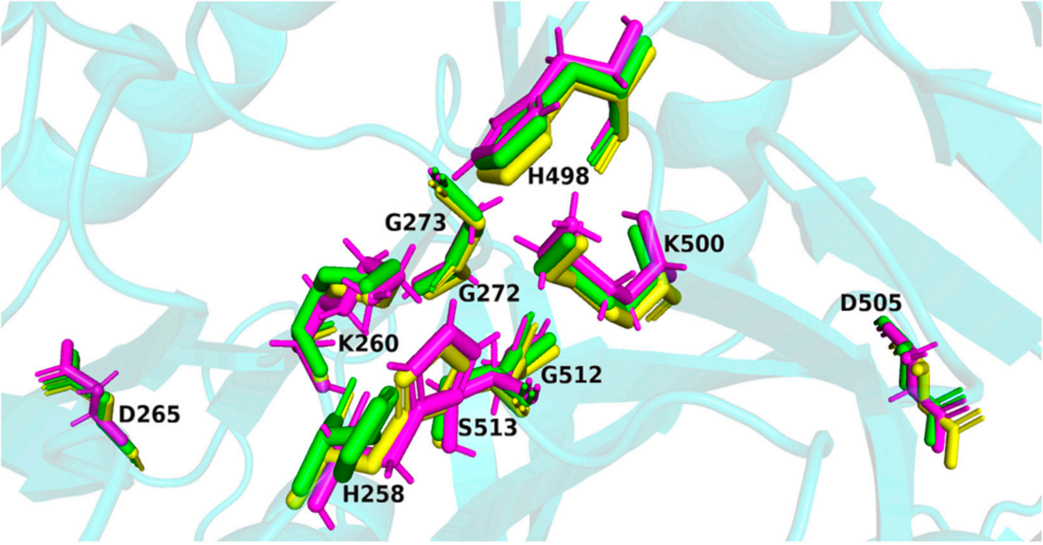

Supplementary Figure S3. The three-dimensional (3D) structure of wild type MsPLD.

(A) Six molecules in the asymmetric unit were shown with different colors. (B) Overall structure of MsPLD. MsPLD was composed by 12 α helixs and 21 β sheets connected by various loops. Two His258 and H498 located at the active site of the protein were shown in sticks. (C) Overall topology of MsPLD. (D) Two classical HxKxxxxD (HKD) motifs were found in MsPLD and showed high structural consistence with several PLDs within the PDB. MsPLD (magentas, PDB ID: 7WU1), *Streptomyces* sp. PMF PLD (yellow, PDB ID: 1F0I), SaPLD (green, PDB ID: 2ZE4). The 3D molecular visualization was performed with PyMOL software.

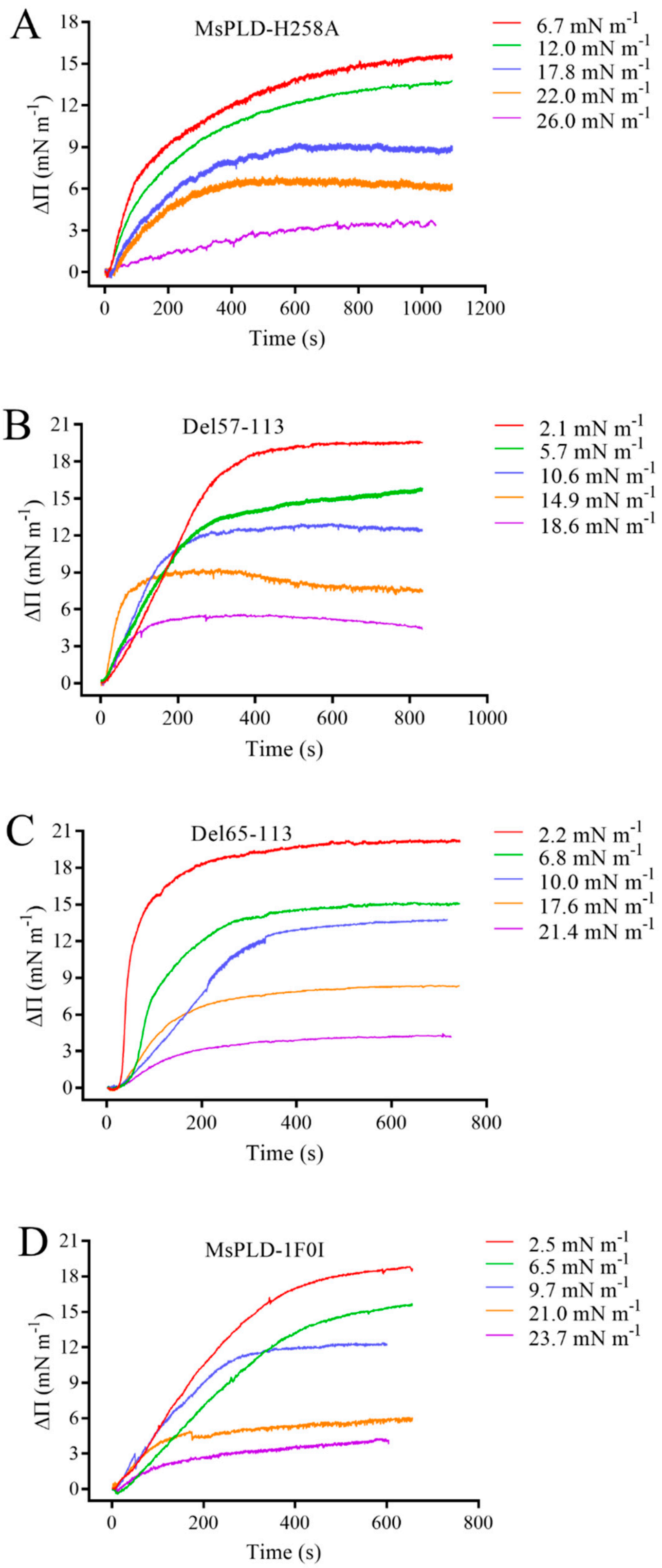

Supplementary Figure S4. Effects of various initial surface pressure (Πi) on the adsorption of wild type MsPLD or its mutants onto PC monolayer. (A) Effects of various initial surface pressure (Πi) on the adsorption of MsPLD-H258A. (B) Effects

of various initial surface pressure (Πi) on the adsorption of Del57-113. (C) Effects of various initial surface pressure (Πi) on the adsorption of Del65-113. (D) Effects of various initial surface pressure (Πi) on the adsorption of MsPLD-1F0I. MsPLD and its corresponding mutants (5.0 μM final concentration) were injected into the aqueous sub-phase of teflon trough, respectively. Surface pressure increase due to the adsorption of the protein onto phospholipid monolayer that spread at initial surface pressures was continuously monitored.

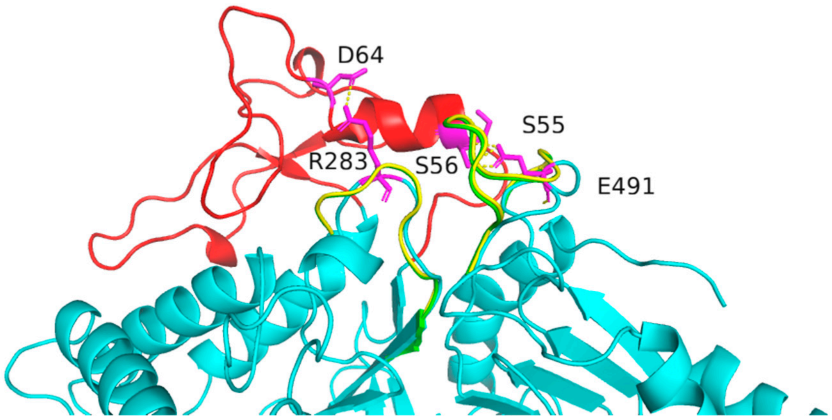

Supplementary Figure S5. Partial superimposition and comparison of the three typical loop segments of MsPLD (cyans, PDB ID: 7WU1) with other *Streptomyces* PLDs. *Streptomyces* sp. PMF PLD (yellow, PDB ID: 1F0I), SaPLD (green, PDB ID: 2ZE4). The extra loop segment solely exist on MsPLD was shown in red. The hydrogen bonds formed with related residues between the adjacent two loops were marked by dashed line in orange. Amino acid residues with interactions on the loops were shown in magentas sticks.
